# Preparation of Polysaccharides from *Ramulus mori*, and Their Antioxidant, Anti-Inflammatory and Antibacterial Activities

**DOI:** 10.3390/molecules24050856

**Published:** 2019-02-28

**Authors:** Wansha Yu, Hu Chen, Zhonghuai Xiang, Ningjia He

**Affiliations:** State Key Laboratory of Silkworm Genome Biology, Southwest University, Beibei, Chongqing 400715, China; yuwansha123456@163.com (W.Y.); c924992@sina.com (H.C.); xbxzh@swu.edu.cn (Z.X.)

**Keywords:** *Ramulus mori*, polysaccharides, bioactivity

## Abstract

The extraction of *Ramulus mori* polysaccharides (RMPs) was optimized using response surface methodology (RSM). The optimal process conditions, which gave the highest yield of RMPs (6.25%) were 80 °C, 50 min, and a solid–liquid ratio of 1:40 (g/mL), with the extraction performed twice. The RMPs contained seven monosaccharides, namely, mannose, rhamnose; glucuronic acid, glucose, xylose, galactose, and arabinose, in a 1.36:2.68:0.46:328.17:1.53:21.80:6.16 molar ratio. The glass transition and melting temperatures of RMPs were 83 and 473 °C, respectively. RMPs were α-polysaccharides and had surfaces that resembled a porous sponge, as observed by scanning electron microscopy. RMPs inhibited the proliferation of *Escherichia coli, Staphylococcus aureus*, and *Pseudomonas aeruginosa* and showed antioxidant activity (assessed by three different methods), although it was generally weaker than that of vitamin C. RMPs showed anti-inflammatory activity in a concentration-dependent manner. This study provides a basis for exploring the potential uses of RMPs.

## 1. Introduction

Mulberry (*Morus alba* L.) is a perennial woody plant with many ecological effects, including improving air quality, protecting of water resources, soil integration, and improving of microclimates [1]. In recent years, mulberry has been used to restore vegetation in the Three Gorges Reservoir Region, control rocky desertification, and reconstruct ecological landscapes in China [2,3]. Mulberry leaves are used to rear the domesticated silkworm, with constant pruning critical for the accumulation of leaves to feed more silkworms. During annual cutting, tons of mulberry branches are discarded and burned, representing a significant waste of resources and causing substantial environmental pollution. Mulberry branch, *Ramulus mori*, is a traditional Chinese medicine. Modern studies have shown that *R. mori* contains flavonoids, polyphenols, alkaloids, polysaccharides, and other active ingredients [4,5,6] that can have curative effects, including diabetes-alleviating and liver-protecting effects [7,8].

Response surface methodology (RSM), is a collection of statistical and mathematical techniques that are effective for developing, improving, and optimizing processes [9] and products [10]. RSM has been widely used to optimize process variables for the extraction of polysaccharides [11], flavonoids [12], alkaloids [13], and saponins [14] from various materials.

Anti-diabetic effects mediated by *R. mori* polysaccharides (RMPs) have long been the focus of studies on these natural products [7,8]. However, the structural characterization, and antibacterial, antioxidant, and anti-inflammatory activities of RMPs have yet to be reported. A basic understanding of the structure and biological activity of RMPs is essential for future applications of these polysaccharides. This study aimed to obtain an optimal extraction method for RMPs and explore the potential value of RMPs in the pharmaceutical and food industries.

## 2. Results and Discussion

### 2.1. Single-Factor Experiments

**Figure 1:**
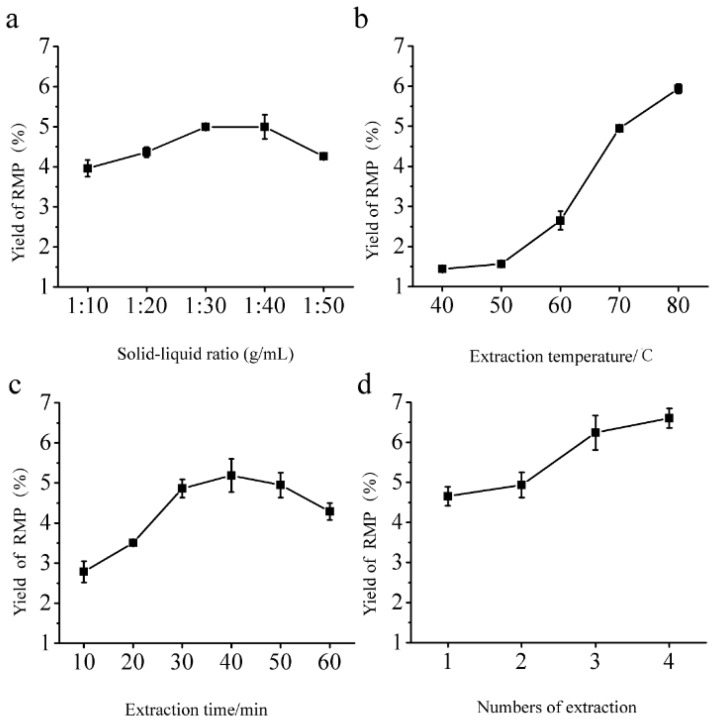
Relationships between (**a**) solid–liquid ratio, (**b**) extraction temperature, (**c**) extraction time, and (**d**) number of extractions and *R. mori* polysaccharide (RMP) yield in single-factor experiments.

Preliminary studies were conducted to investigate the influence of the solid–liquid ratio on the RMP extraction yield when the other extraction parameters were fixed as follows: Extraction temperature, 70 °C; extraction time, 30 min; number of extractions, one. As shown in Figure 1a, the RMP yield increased from 3.96% to 5.00% with an increase in the solid–liquid ratio from 1:10 to 1:40. When the solid–liquid ratio was further increased, the RMP yield decreased to 4.26%. The polysaccharides were rapidly dissolved, which resulted in an increased yield. However, the viscosity of the solvent increased with an increasing solid–liquid ratio, resulting in an increase in the diffusion distance toward the internal tissues [15]. Therefore, the RMP yield increased slowly with increasing solid–liquid ratio, but with a downward trend at the highest solid–liquid ratio (Figure 1a). To achieve increased RMP production using less solvent and in a shorter time, 1:40 was selected as the optimum solid–liquid ratio.

Figure 1b shows the effect of extraction temperature on the RMP yield. Different extraction temperatures were used, with the other extraction parameters fixed as follows: Solid–liquid ratio, 1:40; extraction time, 30 min; number of extractions, one. The RMP yield increased from 1.44% to 5.94% when the extraction temperature was increased from 40 to 80 °C, perhaps due to the higher solubility and diffusivity of the polysaccharides in water at higher temperatures [16]. Accordingly, 80 °C was selected as the optimum extraction temperature.

The effect of different extraction times (10, 20, 30, 40, 50, and 60 min) on the RMP yield was investigated, with the other extraction parameters fixed as follows: Solid–liquid ratio, 1:40; extraction temperature, 80 °C; number of extractions, one. As shown in Figure 1c, the RMP yield increased with increasing extraction time from 10 to 40 min, with RMP production approaching a maximum at 40 min (5.18%) and decreasing thereafter. This decrease indicated that longer extraction times led to thermal instability and degradation of the RMPs [17]. Consequently, 40 min was selected as the optimal RMP extraction times.

The effect of a different number of extractions (1–4) on the RMP yield was investigated, with the other extraction parameters fixed as follows: Solid–liquid ratio, 1:40; extraction temperature, 80 °C; and extraction time, 40 min (Figure 1d). The yield increased as the number of extractions increased from one to four. Therefore, three extractions was selected as the optimum number for subsequent experiments.

### 2.2. Model Fitting and Statistical Analysis

According to these single-factor experiments, the ultrasound assisted extraction variables were established (Table 1). The actual and predictive values of responses (RMP yields) under different conditions are shown in Table 1. The predicted response value (*Y*_RMP_) was based on the following second order polynomial equation:

*Y*_RMP_ = 4.38 + 1.79*X_1_* + 0.24*X_2_* + 0.031*X_3_* + 0.51*X_4_* + 0.38*X_1_X_2_* + 0.35*X_1_X_3_* − 0.56*X_1_X_4_* + 0.14*X_2_X_3_* − 0.23*X_2_X_4_* + 0.23*X_3_X_4_* − 0.47 *X_1_^2^*− 0.33 *X_2_^2^* − 0.50 *X_3_^2^* + 0.073*X_4_^2^* (1)

where *Y*_RMP_ is the RMP yield and *X_1_*, *X_2_, X_3_*_,_ and *X_4_* are the coded variables for extraction temperature, extraction time, solid–liquid ratio, and number of extractions, respectively. From the response surface method, analysis of variance (ANOVA) for the screening test model in Table 2 gave a determination coefficient (*R*^2^) of 0.9997. The coefficient of variation was low, at only 0.83%, indicating that this model (Equation (1)) had a high and reliable degree of precision. As shown in Table 2, the large *F*-value (3356.14) and low *p*-value (<0.0001 **) indicated that this model was accurate.

### 2.3. Optimization of RMP Extraction

**Figure 2:**
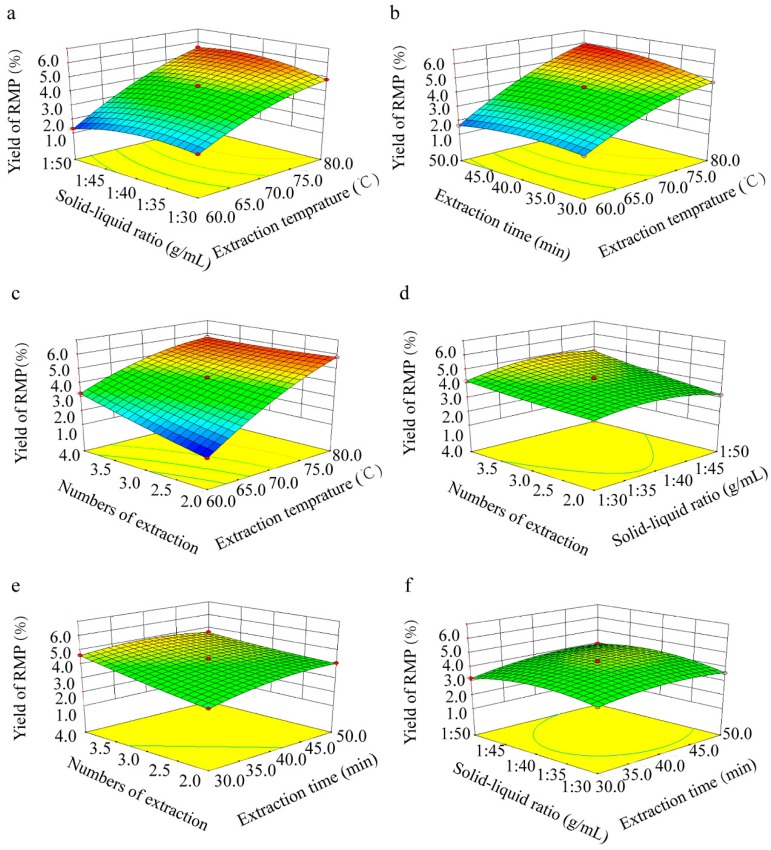
3D response surface plots showing the interaction effects on the RMP extraction yield: (**a**) Solid–liquid ratio and extraction temperature; (**b**) extraction time and extraction temperature; (**c**) number of extractions and extraction temperature; (**d**) number of extractions and solid–liquid ratio; (**e**) number of extractions and extraction time; and (**f**) and solid–liquid ratio and extraction time.

The 3D response surface plots are shown in Figure 2a–f. The optimal values of the tested variables provided the highest RMP yield of 6.37%. The predicted variable parameters were as follows: Extraction temperature, 80 °C; extraction time, 50 min; solid–liquid ratio, 1:42.86 (g/mL); number of extractions, two. However, the solid–liquid ratio was modified to 1:40 (g/mL) in consideration of the actual production process. Under these conditions, the experimental RMP yield was 6.25 ± 0.38% (*n* = 3), which was close to the predicted value (*P* > 0.05). This result validated the response model and the existence of an ideal optimum point.

### 2.4. Identification of Monosaccharides

**Figure 3:**
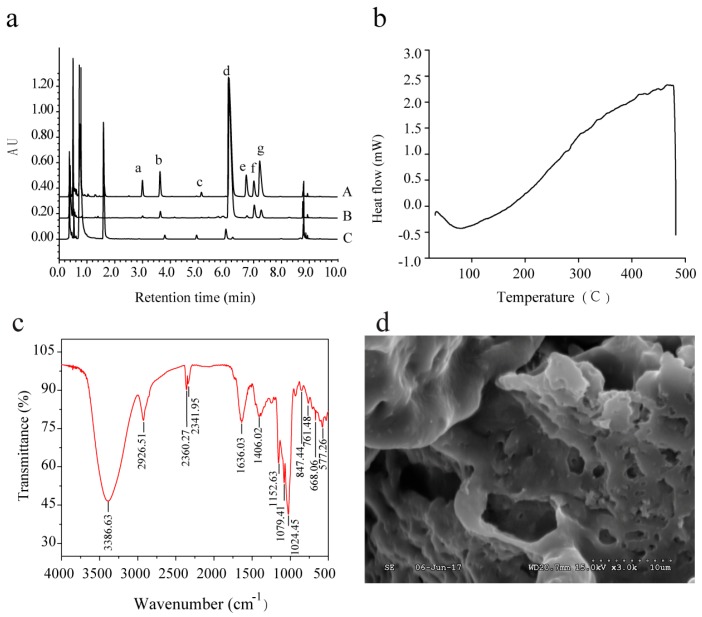
(**a**) Chromatograms of monosaccharide standards and samples (A: monosaccharide standards; B: hydrolyzed RMPs; C: unhydrolyzed RMPs; a: mannose; b: rhamnose; c: glucuronic acid; d: glucose; e; xylose; f: galactose; and g: arabinose); (**b**) DSC thermogram of RMPs; (**c**) FT-IR spectra of RMPs; (**d**) SEM photograph of RMPs (3000×).

The monosaccharide composition of the RMPs was analyzed by ultra-performance liquid chromatography coupled with a tunable ultraviolet detector (UPLC-TUV; Figure 3a). The RMPs consisted of mannose, rhamnose, glucuronic acid, glucose, xylose, galactose, and arabinose at a molar ratio of 1.36:2.68:0.46:328.17:1.53:21.80:6.16 (Table 3).

### 2.5. Differential Scanning Calorimetry (DSC) Analysis

Figure 3b shows the DSC diagram of the RMPs. During the heating process, the solid structure of the RMPs was altered at 83 °C. An exothermic peak was observed at 470 °C, which indicated that the RMPs were in a molten state, and then oxidized and decomposed. Previous studies have suggested that the thermal behavior of polysaccharides is influenced by their chemical composition, physical form, mannose content, and molecular weight [18,19].

### 2.6. FT-IR Spectroscopy Analysis

The IR spectrum of the RMPs (Figure 3c) exhibited bands at 3386.63, 2926.51, and 1406.02 cm^−1^ indicating –OH stretching, C–H stretching, and C–H bending vibrations, respectively, which are characteristic absorption bands of carbohydrates [20]. The band at 847.44 cm^−1^ was characteristic of an α-polysaccharide [21]. Furthermore, absorption peaks at 1024.45, 1079.41, and 1152.63 cm^−1^ suggested the presence of C–O and C–C bands in RMPs [22]. The peaks at 2360.27 cm^−1^ and 2341.95 cm^−1^ were attributed to CO_2_ and H_2_O trapped in the sample, respectively [23].

### 2.7. Morphological Analysis

The surface of the RMPs resembled a rugged sponge (Figure 3d), perhaps due to freeze-drying and water evaporation. Zhu found that the antitumor activity of polysaccharides isolated from *Cordyceps gunnii* differed depending on the extraction method [24]. PPS_MAE_ (polysaccharides from microwave-assisted extraction) had the strongest antitumor activity, perhaps due to the small and thin lamellar structure of PPS_MAE_, such that the tumor cells could be fully exposed to the polysaccharides. Therefore, observing the surface structure of the polysaccharides could provide a scientific basis for the biological activity of RMPs.

### 2.8. Antibacterial Activity of RMPs

**Figure 4:**
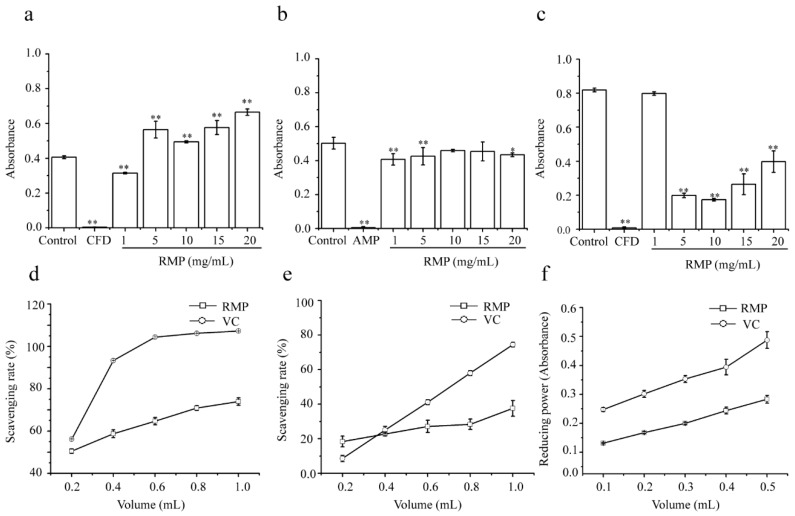
Antibacterial and antioxidant activity of RMPs. Effects of RMPs on the proliferation of (**a**) *E. coli*, (**b**) *S. aureus*, and (**c**) *P. aeruginosa*. (**d**) Hydroxyl radical scavenging activity; (**e**) superoxide-radical scavenging activity; and (**f**) reducing the power of RMPs. Values shown are means ± SD obtained from three measurements. Abbreviations: CFD, Cefobid; AMP, ampicillin; and VC, vitamin C. ** *P* < 0.01 and * *P* < 0.05.

Figure 4a–c illustrate the antimicrobial activity of RMPs against *E. coli*, *S. aureus*, and *P. aeruginosa*. The antibacterial system consisted of three different bacteria (10^5^ CFU/mL) incubated with different RPM concentrations, and the absorbance was measured at 600 nm. The results indicated that Gram-negative bacteria *P. aeruginosa* had the highest sensitivity (*P* < 0.01) to RMPs at the studied concentrations (Figure 4c). The highest bacterial resistance against RMPs was observed for *S. aureus* (Figure 4b). It has been suggested that polysaccharides might change the cell wall and membrane permeability of bacteria, or act as a barrier that inhibits bacterial growth by blocking nutrient import [25]. As shown in Figure 4a, the results for *E. coli*, in which the absorbance increased with increasing RMP concentration, did not support these theories. This might be attributed to the ability of *E. coli* to hydrolyze the RMPs and use the produced monosaccharides as a nutritional source [26].

### 2.9. Antioxidant Activity of RMPs

Antioxidant mechanisms include the suppression of hydroxyl radical generations and the scavenging of generated hydroxyl radicals [27]. As shown in Figure 4e, the scavenging activity of the RMPs solution (1.0 mL) toward hydroxyl radicals reached 73.97%, which was 33% lower than that of VC. The superoxide radical is the most active reactive oxygen species and is involved in many physiological and pathological processes [28]. As shown in Figure 4f, the scavenging effects of RMPs increased as the volume was increased from 0.2 to 1.0 mL. When the volume was 1.0 mL, the scavenging rates of RMPs and VC were 37.61% and 74.50%, respectively. In the reducing-power assay, RMPs reduced Fe^3+^ to Fe^2+^, which was monitored by measuring the formation of Perl’s Prussian blue at 700 nm [29]. Although the reducing power of RMPs at volumes of 0.1–0.5 mL was lower than that of VC, it still reached 0.28 at a volume of 0.5 mL (Figure 4g). According to the results of the above three analyses, RMPs possessed antioxidant activity but were less active than VC. We speculated that the antioxidant activity of RMPs might be related to the high glucose content, although further study is needed to validate this claim.

### 2.10. Effect of RMPs on NO Production Inhibition

**Figure 5:**
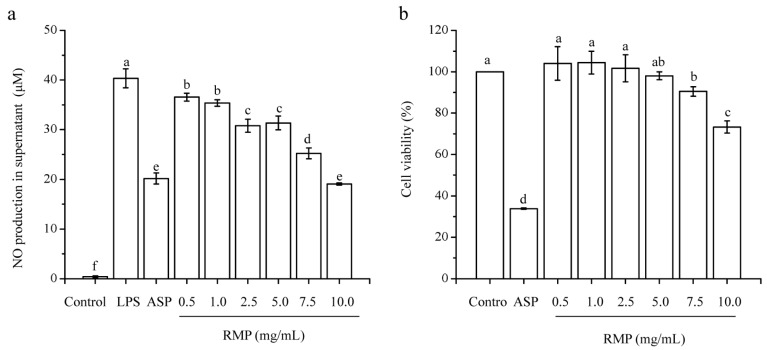
Anti-inflammatory activity of RMPs. (**a**) Effect of RMPs on inhibiting NO production and (**b**) the cell viability of RAW 264.7 cells. Abbreviations: LPS, lipopolysaccharides; ASP, aspirin. Different letters (a-f) indicate significant difference between groups (*P* < 0.05) and same letters indicate *P* > 0.05.

Inflammation is a complex process associated with the immune response. When pathogens invade the human body, endotoxins or cytokines induce macrophages and other cells to express an inducible NO synthase that, through NO generation, plays an important role in the cytotoxicity of activated macrophages and the immunoinflammatory response [30]. Significant inhibition of NO production in a concentration-dependent manner was observed at an RMP concentration of 0.5–10.0 mg/mL (Figure 5a, Table 4). The effect on NO production was even greater than that of the positive control group at an RMP concentration of 10.0 mg/mL. These results indicated that RMPs exhibited antibacterial action and would relieve the inflammatory response caused by infection.

### 2.11. Effect of RMPs on the Cell Viability of RAW 264.7 Cells

RAW 264.7 cells were treated with RMPs at concentrations of 0.5, 1.0, 2.5, 5.0, 7.5, and 10.0 mg/mL with 1.0 μg/mL LPS. The results showed that treatment with RMPs at concentrations of 0.5, 1.0, 2.5, and 5.0 mg/mL had no obvious toxic effect on cell growth compared with the control group. The cell viability of aspirin (1.0 mg/mL) was 33.8%, which was the lowest among all the groups tested. In general, RMPs did not exhibit any toxic effects in the concentration range of 0.5–2.5 mg/mL.

## 3. Materials and Methods

### 3.1. Materials

*R. mori* was obtained from the mulberry breeding center at Southwest University, Chongqing, China. *E. coli*, *P. aeruginosa*, and *S. aureus* were obtained from the Laboratory of Silkworm Pathophysiology and Application of Microbial Research of Southwest University. RAW 264.7 cells were provided by Procell Co., Ltd (Wuhan, China). Aspirin, vitamin C, lipopolysaccharides, and DMSO were purchased from Sigma-Aldrich (St. Louis, MO, USA). Streptomycin, penicillin, fetal bovine serum, trypsin, and Dulbecco’s modified Eagle medium (DMEM) were purchased from Gibco (Grand Island, NY, USA).

### 3.2. Extraction of RMPs

The dry biomass powder (10 g) was extracted with water (400 mL). The mixture was treated with ultrasound for 50 min in a water bath at 80 °C. After being centrifuged at 10,000× *g* for 5 min, the supernatant was collected. The RMP in pellets was extracted again using the same method. The supernatant gathered from two extractions was combined. Four times the volume of 95% ethanol was slowly added to the supernatant and the mixture was stored at 4 °C overnight for sedimentation. The precipitate was collected by centrifugation at 10,000× *g* for 5 min. The coarse RMPs were then washed and deproteinized using the Sevag method. The total carbohydrate content was measured by the phenol–H_2_SO_4_ assay for RMPs [31], using glucose as a standard (*R^2^* = 0.9969).

### 3.3. Experimental Design and Statistical Analysis

The Box–Behnken design (BBD, design expert software, version 8.0.5) was applied to determine the experimental conditions, which combined four independent variables at three levels, namely, extraction temperature (*X_1_*: 60, 70, and 80 °C ), extraction time (*X_2_*: 30, 40, and 50 min), solid–liquid ratio (*X_3_*: 1:30, 1:40, and 1:50), and number of extractions (*X_4_*: 2, 3, and 4). Data were analyzed using a quadratic polynomial model that expressed the response as a function of the independent variables as follows:

(2) $$Y=\;A_{0}+\;\overset{4}{\underset{i=1}{\sum }}A_{i}X_{i}+\overset{4}{\underset{i=1}{\sum }}A_{ii}X_{ii}^{2}\;+\;\overset{3}{\underset{i=1}{\sum }}\overset{4}{\underset{j=i+1}{\sum }}A_{ij}X_{i}X_{j},$$

where *Y* is the value of the studied response predicted by the model, *A*_0_ is a constant coefficient, *A_i_* is the linear coefficient for each independent variable, *A_ii_* is the interaction coefficient, and *X_i_* and *X_j_* are the actual values of the independent variables.

### 3.4. UPLC Analysis of The Monosaccharide Composition of RMPs

According to a literature method [32], RMPs were hydrolyzed into monosaccharides and 1-phenyl-3-methyl-5-pyrazolone (PMP)-labeled monosaccharides were derived for use in the UPLC system. Chromatographic separation was conducted on a Waters Acquity UPLC I-Class system, including a tunable UV detector and an ACQUITY UPLC BEH C_18_ column (1 mm × 100 mm, 1.7 μm, Waters, Milford, MA, USA). The column temperature was set at 40 °C. Gradient elution was conducted by varying the proportion of each mobile phase at a flow rate of 0.17 mL/min. Mobile phase A consisted of 50 mM NH_4_OAc–NH_3_ in H_2_O (pH 9.5) and mobile phase B was acetonitrile. The gradient elution comprised a linear increase from 8% to 15% B over 5.5 min and was then held at 15% B for 2.5 min. The wavelength was 250 nm and the injection volume was 1 μL. Glucose, glucuronic acid, mannose, rhamnose, xylose, galactose, and arabinose with purity greater than 98% were purchased from ChromaBio (Chengdu, China) and used to prepare the standard solution. The correlation coefficients (*R^2^*) and linearity ranges of the seven monosaccharides were as follows: Glucose (*R^2^* = 0.9998, 0–1600 μg/mL), glucuronic acid (*R^2^* = 0.9996, 0–10 μg/mL), mannose (*R^2^* = 0.9998, 0–40 μg/mL), rhamnose (*R^2^* = 0.9999, 0–80 μg/mL), xylose (*R^2^* = 0.9992, 0–80 μg/mL), galactose (*R^2^* = 0.9979, 0–60 μg/mL), and arabinose (*R^2^* = 0.9986, 0–100 μg/mL).

### 3.5. FT-IR Analysis of RMPs

RMPs were identified by Fourier transform infrared spectroscopy (Thermo Scientific, MA, USA) in the frequency range of 4000–500 cm^−1^ using the KBr pressed-disk method. The dried RMPs were mixed with KBr powder and pressed into 1-mm pellets for measurement. Three replicate spectra were obtained.

### 3.6. Antibacterial Experiments In Vitro

Three different bacteria (10^5^ CFU/mL) were inoculated into sterile LB liquid medium, containing RMPs at concentrations of 1, 5, 10, 15, and 20 mg/mL. Ampicillin (AMP) and Cefobid (CFD) were used as positive control groups. The absorbance of the cell concentrations was measured at 600 nm to assess the antibacterial activity of RMPs after incubation for 24 h at 37 °C [26].

### 3.7. Antioxidant Activity of RMPs

Three different methods were used to analyze the antioxidant activity of RMPs, namely, a hydroxyl radical scavenging assay, a superoxide-radical scavenging assay, and a reducing-power assay. The hydroxyl radical scavenging activity, superoxide radical scavenging activity, and reducing-power of the RMPs were determined using a previously reported method with slight modification [33,34,35]. Vitamin C (V_C_) was diluted in deionized water and used as a positive control in the above three experiments.

### 3.8. Determination of Anti-Inflammatory Activity

The cell viability was analyzed using a CKK-8 assay in vitro. RAW 264.7 cells were seeded into 96-well culture plates (10^5^ cells/well) and incubated at 37 °C with 5% CO_2_ for 24 h. Cells were exposed to the culture medium containing RMPs at concentrations of 0.5, 1.0, 2.5, 5.0, 7.5, and 10 mg/mL. After incubation for 24 h, 10 μL of CKK-8 solution was added to each well. The absorbance was then detected at 450 nm after incubation at 37 °C for 1 h. RAW 264.7 cells (10^5^ cells/mL) were plated in 96-well plates and subsequently treated with lipopolysaccharides (LPS, 10^3^ ng/mL) in the presence of different RMPs concentrations (0.5, 1.0, 2.5, 5.0, 7.5, and 10 mg/mL) for 24 h. Aspirin (ASP) was used as a positive control. The supernatant of each culture (50 μL) was mixed with Griess reagent (100 μL) and then the amount of NO production was determined.

## 4. Conclusions

RSM was applied to optimize the RMP extraction conditions. The optimum conditions for maximum biomass in RMP production were a solid–liquid ratio of 1:40 (g/mL), 80 °C, 50 min, with extraction performed twice. The RMPs contained seven monosaccharides, namely, mannose, rhamnose, glucuronic acid, glucose, xylose, galactose, and arabinose with molar a ratio of 1.36:2.68:0.46:328.17:1.53:21.80:6.16. The RMPs were α-polysaccharides with characteristic absorption bands of carbohydrates and a loose porous sponge-like surface. The RMPs showed significant antibacterial, antioxidant, and anti-inflammatory activities. This study provides a basis for exploring the potential uses of RMPs.

## Figures and Tables

**Table 1:** Experimental design with predicted and experimental extraction yield of RMPs.

Independent Variables					Symbol	Range and Level		
						−1	0	+1
Extraction temperature (°C)					*X_1_*	60	70	80
Extraction time (min)					*X_2_*	30	40	50
Solid-liquid ratio (g/mL)					*X_3_*	1:30	1:40	1:50
Numbers of extraction					*X_4_*	2	3	4
**Run**	**Coded Variable Levels**				**Extraction Yield (%)**			
	***X_1_***	***X_2_***	***X_3_***	***X_4_***	**Experimental**		**Predicted**	
1	60	30	1:40	3	1.91		1.93	
2	80	30	1:40	3	4.74		4.75	
3	60	50	1:40	3	1.63		1.65	
4	80	50	1:40	3	5.97		5.99	
5	70	40	1:30	2	3.63		3.63	
6	70	40	1:50	2	3.22		3.24	
7	70	40	1:30	4	4.20		4.21	
8	70	40	1:50	4	4.69		4.72	
9	60	40	1:40	2	1.12		1.12	
10	80	40	1:40	2	5.81		5.82	
11	60	40	1:40	4	3.27		3.26	
12	80	40	1:40	4	5.73		5.73	
13	70	30	1:30	3	3.41		3.42	
14	70	50	1:30	3	3.59		3.61	
15	70	30	1:50	3	3.22		3.20	
16	70	50	1:50	3	3.97		3.96	
17	60	40	1:30	3	1.95		1.93	
18	80	40	1:30	3	4.85		4.82	
19	60	40	1:50	3	1.31		1.30	
20	80	40	1:50	3	5.59		5.58	
21	70	30	1:40	2	3.15		3.14	
22	70	50	1:40	2	4.10		4.08	
23	70	30	1:40	4	4.64		4.63	
24	70	50	1:40	4	4.67		4.65	
25	70	40	1:40	3	4.42		4.38	
26	70	40	1:40	3	4.39		4.38	
27	70	40	1:40	3	4.37		4.38	
28	70	40	1:40	3	4.41		4.38	
29	70	40	1:40	3	4.31		4.38	

**Table 2:** Analysis of variance for the response surface regression model.

Source	Sum of Squares	df	Mean Square	*F*	*P*
Model	48.41	14	3.46	3356.14	<0.0001 **
*X*_1_	38.52	1	38.52	37385.9	<0.0001 **
*X*_2_	0.68	1	0.68	661.55	<0.0001 **
*X*_3_	0.011	1	0.011	11.07	0.0050 **
*X*_4_	3.17	1	3.17	3078.94	<0.0001 **
*X*_1_X_2_	0.57	1	0.57	553.23	<0.0001 **
*X*_1_X_3_	0.48	1	0.48	462.07	<0.0001 **
*X*_1_X_4_	1.24	1	1.24	1206.6	<0.0001 **
*X*_2_X_3_	0.081	1	0.081	78.83	<0.0001 **
*X*_2_X_4_	0.21	1	0.21	205.37	<0.0001 **
*X*_3_X_4_	0.2	1	0.2	196.53	<0.0001 **
*X*_1_^2^	1.44	1	1.44	1393.12	<0.0001 **
*X*_2_^2^	0.71	1	0.71	687.3	<0.0001 **
*X*_3_^2^	1.63	1	1.63	1584.35	<0.0001 **
*X*_4_^2^	0.035	1	0.035	33.86	<0.0001 **
Residual	0.014	14	0.001		
Lack of fit	0.0068	10	0.0068	0.36	0.9138
Pure error	0.0076	4	0.0019		
Cor. total	48.43	28			
*R*^2^ = 0.9997; *R*^2^_adj_ = 0.9994; *R*^2^_pred_ = 0.9989; *R*_SN_ = 210.828; CV = 0.83%					

** *P* < 0.01.

**Table 3:** Chromatography information and contents of monosaccharide.

Monosaccharide	Regression Equations	*R^2^*	Sample Hydrolyzed (μg/mL)	Sample Unhydrolyzed (μg/mL)	RMPs (μg/mL)
a-Mannose	*Y* = 1.53e + 0.04*X* − 7.38	0.9998	2.79	0.34	2.45
b-Rhamnose	*Y* = 1.29e + 0.04*X* + 1.62	0.9999	12.07	7.67	4.40
c-Glucuronic Acid	*Y* = 1.13e + 0.04*X* + 1.25	0.9996	1.15	0.25	0.90
d- Glucose	*Y* = 1.31e + 0.04*X* + 1.44	0.9998	593.59	2.88	590.71
e-Xylose	*Y* = 3.08e + 0.04*X* − 6.61	0.9992	2.69	0.40	2.29
f-Galactose	*Y* = 1.09e + 0.04*X* − 7.97	0.9979	40.33	1.05	39.28
g-Arabinose	*Y* = 3.30e + 0.04*X* − 2.12	0.9986	9.52	ND	9.52

Means and standard deviations are based on three replicates. ND: Not detected.

**Table 4:** Anti-inflammatory activity of RMPs.

Group	NO Production (μM)	Cell Viability (%)
Control	0.40 ± 0.17 ^f^	100.00 ± 0.00 ^a^
LPS	40.36 ± 1.92 ^a^	—
ASP 1.0 mg/mL	20.14 ± 1.12 ^e^	33.80 ± 0.32 ^d^
RMP 0.5 mg/mL	36.56 ± 0.77 ^b^	104.02 ± 8.10 ^a^
RMP 1.0 mg/mL	35.38 ± 0.65 ^b^	104.42 ± 5.48 ^a^
RMP 2.5 mg/mL	30.79 ± 1.33 ^c^	101.68 ± 6.54 ^a^
RMP 5.0 mg/mL	31.36 ± 1.37 ^c^	98.03 ± 1.88 ^a,b^
RMP 7.5 mg/mL	25.23 ± 1.09 ^d^	90.52 ± 2.28 ^b^
RMP 10.0 mg/mL	19.03 ± 0.22 ^e^	73.29 ± 2.91 ^c^

Same letter means *P* > 0.05; different letters mean *P* < 0.05. Abbreviations: LPS, lipopolysaccharides; ASP, aspirin.
